# Microbiological testing of clinical samples before and after periodontal treatment. A comparative methodological study between real‐time PCR and real‐time‐PCR associated to propidium monoazide

**DOI:** 10.1002/cre2.464

**Published:** 2021-07-03

**Authors:** Maria Sereti, Alkisti Zekeridou, Jose Cancela, Andrea Mombelli, Catherine Giannopoulou

**Affiliations:** ^1^ Division of Regenerative Dentistry and Periodontology University Clinics of Dental Medicine, University of Geneva Geneva Switzerland

**Keywords:** plaque, bacteria, RT‐PCR, propidium monoazide, periodontal treatment

## Abstract

**Objectives:**

The aim of the present methodological study was to evaluate the discrepancies in the detection of a number of periodontally involved pathogenic bacteria obtained from clinical samples by two methods: the quantitative Polymerase Chain Reaction (qPCR) and the qPCR combined with pre‐treatment by Propidium Monoazide (PMA).

**Material and methods:**

Plaque and saliva samples were obtained from 30 subjects: 20 subjects with chronic or aggressive periodontitis in need of periodontal therapy with or without antibiotics and 10 subjects in Supportive Periodontal Treatment (SPT). The clinical samples taken before treatment (BL) and 1 month later (M1), were divided in two aliquots: one was immediately treated with PMA while the other was left untreated. All samples were further analyzed with qPCR after DNA extraction, for the detection of *Aggregatibacter actinomycetemcomitans* (Aa), *Porphyromonas gingivalis* (Pg), *Tannerella forsythia* (Tf), *Treponema denticola* (Td), *Parvimonas micra* (Pm), and *Prevotella intermedia* (Pi).

**Results:**

Large inter‐individual variations were observed in the concentration of the studied bacteria. At both instances (BL and M1) and for the three groups, significantly lower counts of bacteria were depicted when plaque and saliva samples were pre‐treated with PMA as compared to those without treatment. Treatment resulted in significant decreases in the number of bacteria, mainly in the plaque samples. However, these changes were almost similar in the three groups independently of the method of detection used (PMA‐qPCR vs. q‐PCR).

**Conclusion:**

Removal of DNA from non‐viable cells with PMA treatment is an easily applied step added to the classical qPCR that could give accurate information on the presence of viable bacterial load and evaluate the response to periodontal treatment.

## 1 INTRODUCTION

For the detection and quantification of microbial pathogens in clinical specimens, culture techniques have long been considered the gold standard. In dental research, clinical trials used these methods extensively to evaluate antimicrobial effects of periodontal therapy (Loomer, 2004). Culture techniques were found to be fairly reproducible and consistent in demonstrating reductions of bacterial counts after various types of periodontal treatment (Mombelli et al., 1989). However, thorough analyses required advanced technical skills and specific equipment to assure the survival and growth of the microorganisms in vitro. Anaerobic bacteria that were thought to play important roles in periodontal diseases (Haffajee & Socransky, 1994) were especially difficult to cultivate. Bacteria that could not be grown under laboratory conditions were ignored (Loesche et al., 1992).

More recently, molecular techniques have been developed for the detection and quantification of pathogenic bacteria in oral samples. Among these, the Quantitative Polymerase Chain Reaction (qPCR) is a rapid method with high sensitivity and specificity, allowing the simultaneous detection and quantification of multiple bacterial species at the same time. However, one of its major disadvantages is the inability to differentiate viable from dead cells. This means that after cell death, the DNA still persists and may even serve as a template for PCR amplification for up to 1–2 years (Brundin et al., 2010).

Contrary to classical reports (van Winkelhoff & Winkel, 2005), several studies using qPCR, failed to show significant differences in the bacterial counts before and after periodontal therapy despite the improvement of clinical parameters (Cionca et al., 2010; Mombelli et al., 2017), or after supportive periodontal therapy (Moëne et al., 2010; Müller Campanile et al., 2015), independently of the clinical results. One of the reasons, could be that this technique doesn't discriminate between viable and dead bacteria.

Recently, an adjunct to qPCR analysis has come to light (Nocker et al., 2009). Propidium Monoazide (PMA) is a DNA‐modifying dye that has the ability to intercalate with DNA of cells with compromised membrane (dead or damaged) and thus inhibiting its amplification during qPCR. The benefit of PMA treatment prior to qPCR is the possibility of selective detection and quantification of the viable forms of microorganisms (Nocker et al., 2007).

This technique has already been used in projects regarding food safety (milk, yogurt) (Yu et al., 2017), for environmental testing (water, soil) (Scaturro et al., 2016) and even for evaluation of bacterial and fungal communities on surfaces in the International Space Station (Checinska Sielaff et al., 2019). In the field of dentistry, the combination of qPCR and PMA has been tested in a few in vitro studies demonstrating the efficiency of PMA for differentiating viable and dead oral pathogens (Loozen et al., 2011; Sanchez et al., 2013, 2014) as well as in a small number of in vivo studies investigating for example the efficacy of a mouthwash (Exterkate et al., 2015) or the presence of bacteria in root canal infection (Kim et al., 2013). These few studies suggested that the capacity of the method to distinguish viable from dead bacteria, could help to evaluate more accurately various treatment protocols, with or without antimicrobials.

Thus, the aim of the present methodological study was to evaluate the discrepancies in the results on a number of periodontally involved pathogenic bacteria obtained from clinical samples by two methods: the qPCR alone and the combination of qPCR and PMA.

## 2 MATERIALS AND METHODS

### 2.1 Study design

The study was approved by the Ethical Committee of the University Hospitals of Geneva (Protocol Number 2008‐00420). It is a single center study of 1 month duration involving samples of 30 patients attending the Division of Regenerative Dentistry and Periodontology of the University of Geneva for treatment of periodontitis (*n* = 20) and in maintenance care (*n* = 10). The clinical samples were obtained from subjects under three clinical situations. The first and second groups included chronic or aggressive periodontitis patients in need of active periodontal treatment by means of non‐surgical periodontal therapy either alone or with adjunction of systemic antibiotics, respectively. The third group included subjects that were previously treated for periodontitis and presented persistence of sites with probing pocket depths (PPDs) >4 mm and bleeding on probing (BOP). Although the diagnosis of the cases and the treatment plan was established before the introduction of the new classification on periodontal/periimplant diseases and conditions, according to the new classification, the subjects included presented a periodontitis stage III/IV, grade A‐C (Papapanou et al., 2018; Tonetti et al., 2018). Furthermore, antibiotics were given, following the clinic protocol to specific cases, based on the clinical status and not on microbiological testing. The presence of selected periodontal pathogens was analyzed in saliva and plaque samples by the two techniques, before and 1 month after periodontal therapy.

The inclusion criteria for all subjects were: signed informed consent and age between 18–80 years. Subjects with chronic or aggressive periodontitis presented at least 4 teeth with a PPD ≥6 mm and BOP, clinical attachment loss (CAL) ≥2 mm and radiographic evidence of bone loss. Subjects in maintenance care had completed periodontal therapy not less than 3 months before, and presented at least 1 tooth with PPD ≥5 mm and BOP. Exclusion criteria for the three groups were: no use of antiseptic mouthwashes during the last 2 weeks prior to treatment, no use of systemic antibiotics within the previous 3 months and no need for prophylactic administration of antibiotics.

### 2.2 Study schedule

Three visits were planned for the subjects participating in the study. During the first visit (pre‐baseline) patients signed the consent form and medical history, demographics and medication were obtained. A periodontal examination including PPD and BOP was performed and the study sites were determined: each of the periodontitis patient contributed with 1 site with PPD ≥6 mm, whereas subjects in maintenance care contributed with 1 site with PPD ≥5 mm (Visit 1). During the following visit within 2–4 weeks (Visit 2), saliva and subgingival plaque samples were collected and then each participant was treated depending on his/her needs; either with scaling and root planning with or without antibiotics for the active treatment or with ultrasonic scaling for maintenance. Subjects were recalled after 1 month (Visit 3) for subgingival plaque and saliva collection.

Saliva was obtained at least 1.5 h after eating and brushing, by spitting twice within 1 min into a sterile 1.5‐ml plastic tube. After thorough mixing, two 100 μl samples were obtained: one for treatment with PMA while the other was left untreated. Subgingival plaque was collected from the pre‐determined study sites with two sterile paper points (Dentsply 0.4 mm, diameter, No 40) inserted to the bottom of each pocket and left in situ for 10 s. The pooled samples were placed into 100μl PBS‐containing Eppendorf tubes and immediately transferred to the Division's laboratory for further treatment. The overall design resulted in a total of 60 plaque samples and 60 saliva samples which were further divided in 2 aliquots, one for treatment with PMA while the other was left untreated.

### 2.3 Laboratory procedures

Subgingival plaque and saliva aliquots were immediately treated with a concentration of 100 μM PMA (Biotium, San Francisco, CA, USA) and exposed to light to cross‐link PMA to DNA, according to the manufacturer's protocol.

Then, for all samples (treated and untreated) the genomic DNA was extracted with the GenElute Bacterial Genomic DNA kit (Sigman‐Aldrich Co., St. Louis, MO, USA) according to the manufacturer's protocol. Samples were directly stored at −80°C.

The day of the analysis, quantitative real‐time PCR (RT‐qPCR) was performed in both PMA‐treated and non‐treated samples using species‐specific primers (Kozarov et al., 2006; Shelburne et al., 2000) in order to detect and quantify the six following periodontal pathogens: *Aggregatibacter actinomycetemcomitans* (Aa), *Porphyromonas gingivalis* (Pg), *Tannerella forsythia* (Tf), *Treponema denticola* (Td), *Parvimonas micra* (Pm), and *Prevotella intermedia* (Pi). A SYBR Green dye (Sigman‐Aldrich Co., St. Louis, MO, USA) was used as nucleid acid stain. The RT‐qPCR procedure was carried out by an ABI Prism® 7900HT Sequence detection system (Applied Biosystems, Foster City, CA, USA). Bacterial counts were calculated by comparing with homologous reference. As such, standard curves were realized by using different concentrations of an originally known quantity of each bacteria.

### 2.4 Statistical analysis

To test for a significant difference in bacteria count between the two methods, Mann‐Whitney U tests were conducted for each bacteria type (5x) within each group (3X) and for both saliva and plaque samples (2X) at each time point (2X), for a total of 60 tests. We used the Wilcoxon matched‐pairs signed‐ranks test to analyze the longitudinal changes obtained by each method from baseline (BL) to month 1 (M1). The correlation between saliva and plaque bacterial concentrations were evaluated with the Pearson correlation analysis. The statistical software IBM SPSS Statistics 26 was used for calculations and *p* values of <0.05 were considered statistically significant.

## 3 RESULTS

Twenty subjects with periodontitis and 10 subjects in maintenance care participated in the study. Table 1 displays the baseline characteristics of the participants in the three groups. The study sites had a mean PPD of 8.1 *±* 1.1 mm in Group 1, 9 *±* 1.4 mm in Group 2 and 6 *±* 1.3 mm in Group 3. BOP of all study sites was recorded only for participants in Group 3. The mean number of pockets > 4 mm with BOP in each group, were of 28.55 *±* 18.7, 43.05 *±* 31.2, and 6.95 *±* 6.5, respectively.

**TABLE 1:** Baseline characteristics of the subjects

	Group 1 (*N* = 10)	Group 2 (*N* = 10)	Group 3 (*N* = 10)
Gender			
Male	5	7	6
Female	5	3	4
Age (years), mean *±* SD	51.8 *±* 7.3	46.7 *±* 9.4	65.2 *±* 8.5
BOP+ (number of study sites)	6/10	7/10	10/10
PD, mean ± SD (for study sites)	8.1 *±* 1.1	9 *±* 1.4	6 *±* 1.3
Number of sites PD >4 mm + BOP, mean *±* SD	28.55 *±* 18.7	43.05 *±* 31.2	6.95 *±* 6.5

**FIGURE 1:**
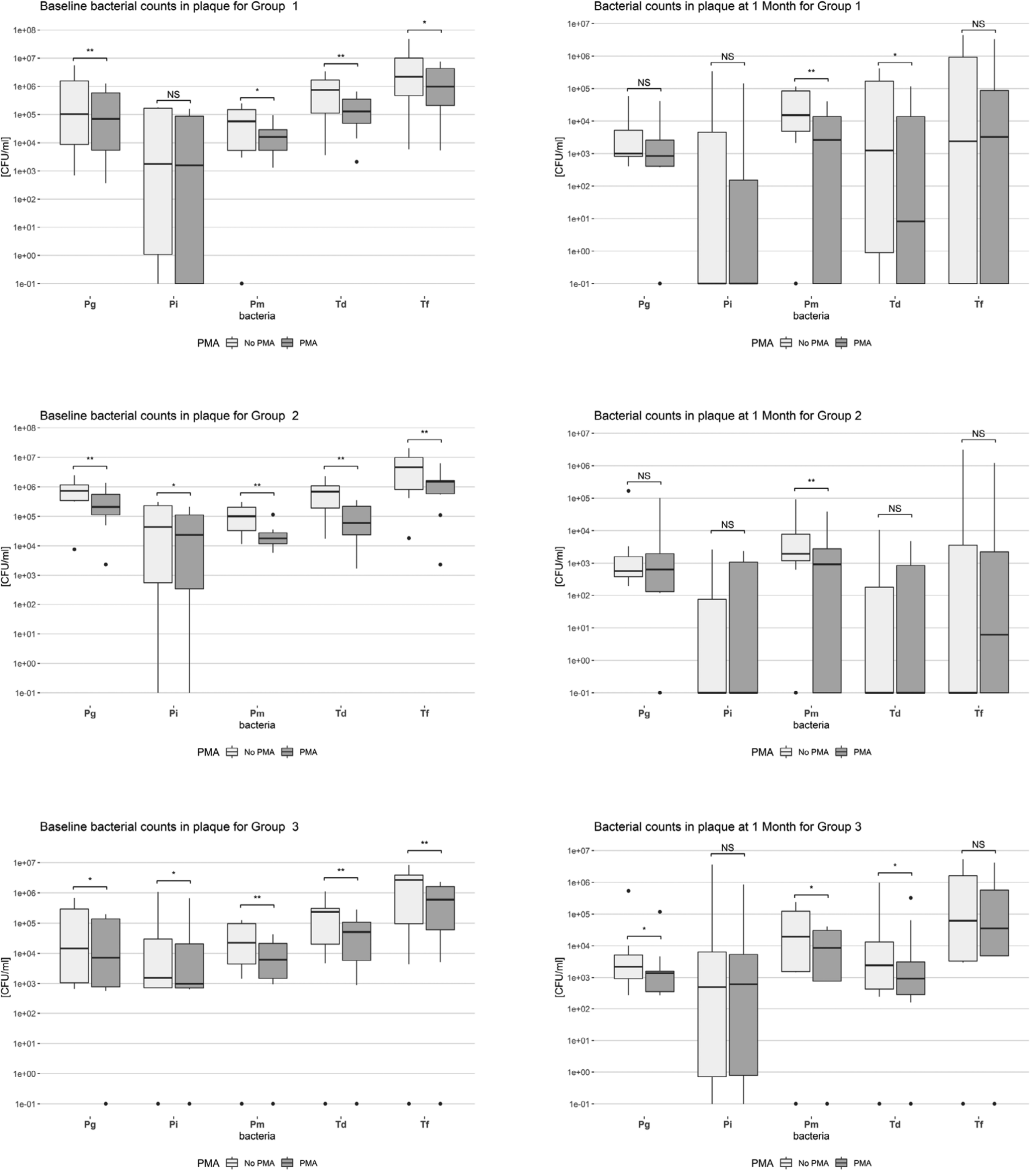
Box‐plots of baseline and 1 month log‐transformed bacterial counts in plaque samples for Group 1 (*N* = 10, SRP), Group 2 (*N* = 10, SRP + AB) and Group 3 (*N* = 10, maintenance care), *p < 0.05, **p < 0.01

Figure 1 shows the box plots of the counts of each studied microorganism with and without PMA in plaque samples for the 3 groups at baseline and 1 month. For each bacteria, we compared the 2 methods (q‐PCR vs. q‐PCR+PMA) for each time point. *A. actinomycetemcomitans* is not displayed because it was never detected at levels exceeding >1000 cells/ml. At BL, when comparing the 2 methods, significantly less amounts of bacteria were found in samples pre‐treated with PMA from all the 3 groups (with the exception of Pi in samples of Group 1). At M1, less differences were observed between the 2 methods: for the 3 groups, only Pm was significantly lower when samples were treated with PMA, whereas Td levels were significantly lower in Groups 1 and 3 and Pg only in Group 3.

**FIGURE 2:**
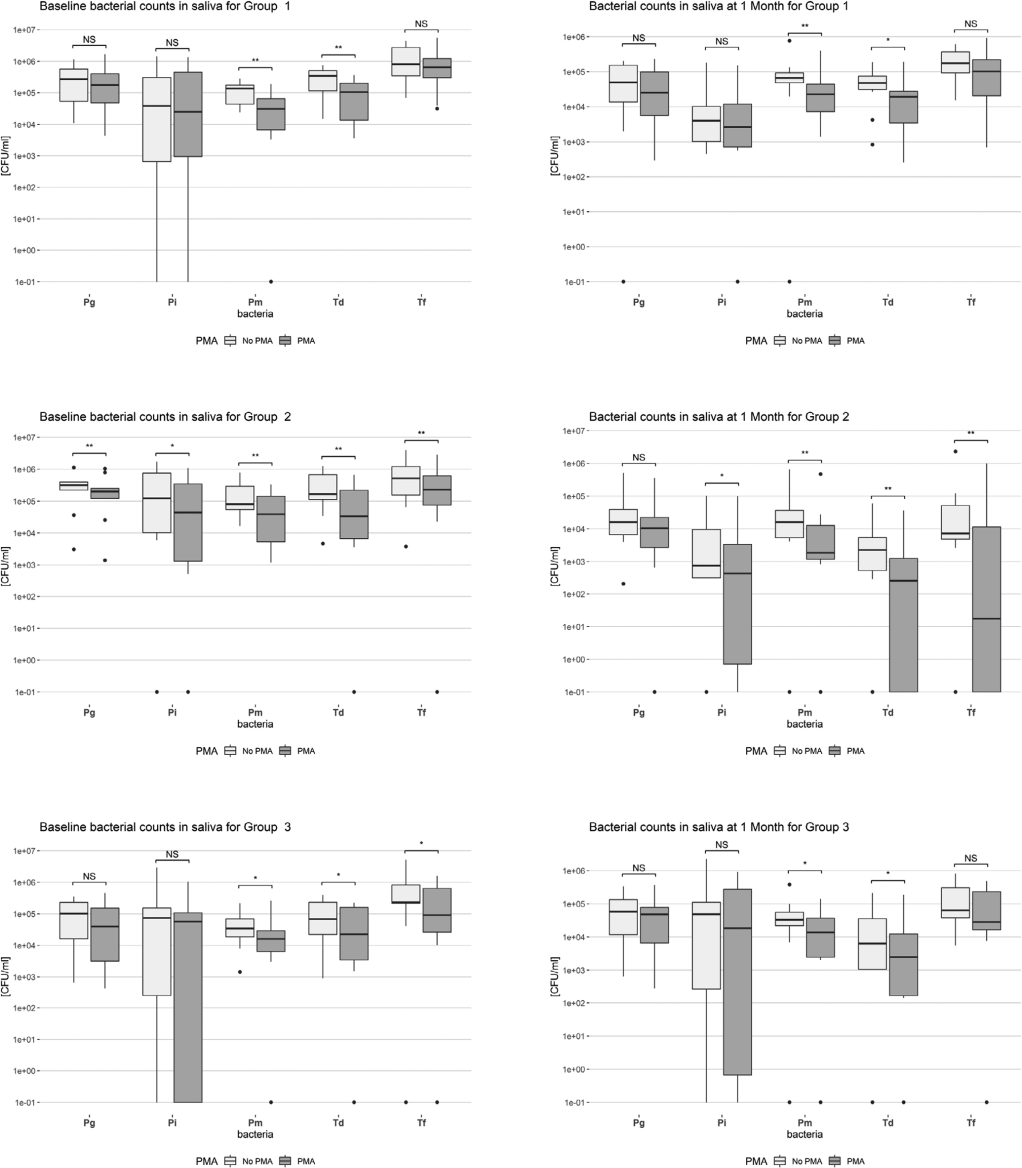
Box‐plots of baseline and 1 month log‐transformed bacterial counts in saliva samples for Group 1 (*N* = 10, SRP), Group 2 (*N* = 10, SRP + AB) and Group 3 (*N* = 10, maintenance care), *p < 0.05, **p < 0.01

Similarly, Figure 2 shows the box plots of the counts with and without PMA of each studied microorganism in saliva samples for the three groups at baseline and 1 month. Td and Pm were significantly lower in samples of all groups when pre‐treated with PMA at both BL and M1. Furthermore, in Group 2, Tf and Pi were significantly lower in the PMA‐treated samples in both time points and Pg only at BL.

**FIGURE 3:**
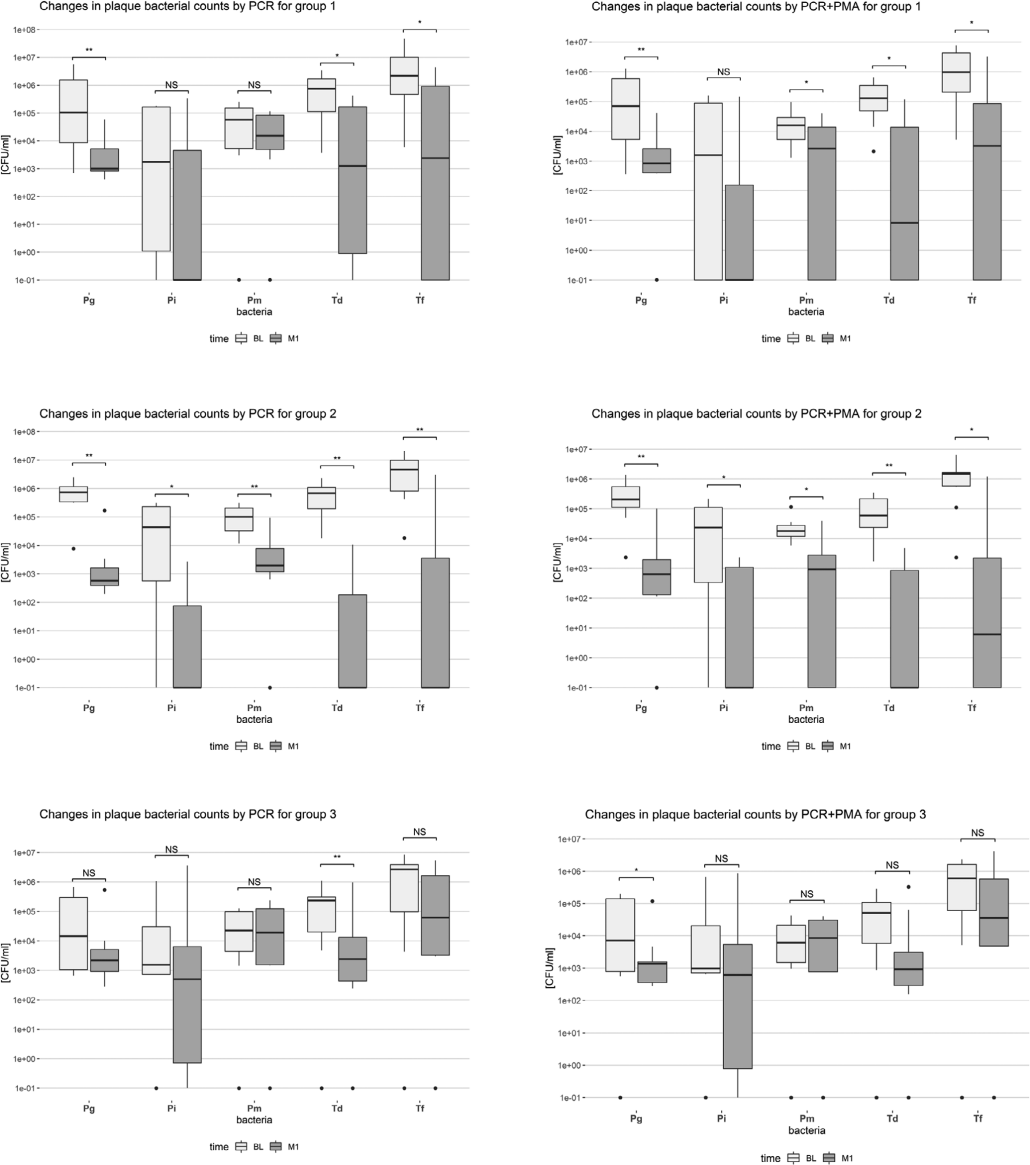
Box‐plots of changes in plaque bacterial counts between Baseline (BL) and 1 Month (M1) by PCR or PCR+PMA for Group 1 (*N* = 10, SRP), Group 2 (*N* = 10, SRP + AB) and Group 3 (*N* = 10, maintenance care), *p < 0.05, **p < 0.01

Figure 3 shows the box plots of changes in plaque bacterial counts between BL and M1 with and without PMA. After periodontal treatment, the levels of all 5 bacteria decreased significantly in Groups 1 and 2 with both methods (except of Pm and Pi in the non‐PMA‐treated samples of Group 1 and of Pi in the Pma‐treated samples for Group 1). In Group 3, the only significant changes from BL to M1 were for Td in the non‐PMA treated samples and for Pg in the PMA‐treated samples.

**FIGURE 4:**
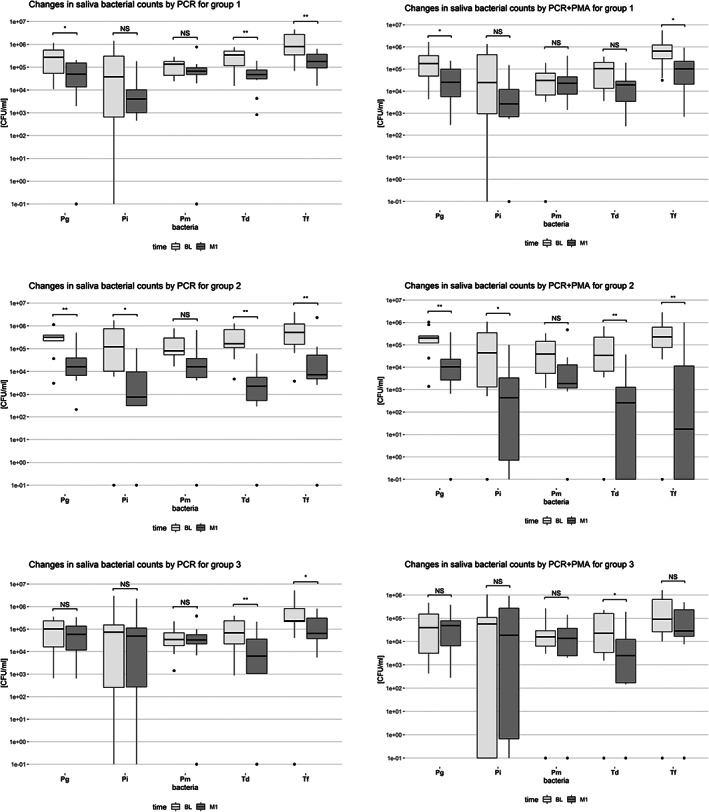
Box‐plots of changes in saliva bacterial counts between Baseline (BL) and 1 Month (M1) by PCR or PCR+PMA for Group 1 (*N* = 10, SRP), Group 2 (*N* = 10, SRP + AB) and Group 3 (*N* = 10, maintenance care), *p < 0.05, **p < 0.01

Similarly, Figure 4 shows the box plots of changes in saliva bacterial counts between BL and M1 with and without PMA After periodontal treatment, the changes of the levels of the studied microorganisms were almost similar in the 3 groups: Td and Tf decreased significantly in all groups (except of Td in the PMA‐treated samples of Group 1 and Tf in PMA‐treated samples of Group 3). Furthermore, independently of the method of detection used, Pg decreased significantly in Groups 1 and 2, whereas Pi decreased only in Group 2.

Finally, Table 2 shows the correlation on the levels of the 5 microorganisms in plaque and saliva as detected by the 2 methods at BL and M1. When q‐PCR was used alone, significant associations were found for all studied bacteria at BL and for Td at M1. When q‐PCR was combined with PMA, significant associations were found for Pg at both time points and for Pm at BL.

**TABLE 2:** Correlation between plaque and saliva levels (with and without PMA) of the 5 microorganisms at baseline and 1 month

Time point	Td/Pl‐Td/S	Pg/Pl‐Pg/S	Pm/Pl‐Pm/S	Tf/Pl‐Tf/S	Pi/Pl‐Pi/S
BL qPCR	***p* = 0.001**	***p* = 0.001**	***p* = 0.000**	***p* = 0.003**	***p* = 0.001**
BL qPCR+PMA	*p* = 0.210	***p* = 0.001**	***p* = 0.004**	*p* = 0.200	*p* = 0.564
M1 qPCR	***p* = 0.013**	*p* = 0.049	*p* = 0.206	*p* = 0.091	*p* = 0.471
M1 qPCR+PMA	*p* = 0.066	***p* = 0.004**	*p* = 0.523	*p* = 0.883	*p* = 0.213

Abbreviations: BL, Baseline; M1, Month 1; Pg, *Porphyromonas gingivalis*; Pi, *Prevotella intermedia*; Pl, Plaque; Pm, *Parvimonas micra*; S, Saliva; Td, *Treponema denticola*; Tf*, Tannerella forsythia*. Level of significance *p < 0.05*.

## 4 DISCUSSION

This was a methodological study for optimized microbiological analysis of clinical samples before and after periodontal therapy. We compared conventional quantitative real‐time PCR (qPCR) and PMA‐qPCR for the detection of 5 periodontal pathogens in plaque and saliva samples of subjects in 3 clinical situations. Furthermore, the microbiological results obtained by the 2 methods were compared between the plaque and saliva samples. The last decades, saliva is considered the preferred oral sample, as it is an easy, quick and non‐invasive way to obtain material containing oral bacteria from various locations including mucosal surfaces, supra‐ and sub‐gingival plaque. The salivary microbiota has been proposed as diagnostic marker for several pathological oral conditions, such as oral cancer, periodontal disease, and dental caries. We found that the concentrations of the studied bacteria varied considerably between the subjects. However, even considering the large inter‐individual variations, we found that there was a significant difference between the results obtained by PMA‐qPCR and q‐PCR alone. At both instances lower counts of bacteria were depicted when samples were pre‐treated with PMA. We may assume that these differences were due to the fact that PMA treatment enables the discrimination between live and dead cells in accordance with other studies both in vitro (Lin et al., 2011; Loozen et al., 2011) and in vivo (Kim et al., 2013). Contrary to PCR which can detect DNA from both viable and dead bacterial cells, leading to overestimating the number of live cells, PMA treatment followed by q‐PCR, can inhibit DNA amplification from dead cells, without affecting the DNA from viable cells. The early study by Moore et al. (1982) based on culture of plaque samples of different maturation stage, reported that there is no evidence that viable bacteria counts increased even though the complexity of the flora increased with time. This further indicates that many bacteria cells found in plaque could be dead and consist a source of antigens and irritants. When qPCR combined with PMA was used in examining biofilms of five oral bacteria after the use of antiseptics, promising results in terms of definition of the mortality of the microorganisms were reported (Alvarez et al., 2013). The study of Exterkate et al. (2015) aimed to evaluate the use of PMA when measuring composition changes in clinical samples from saliva, tongue and plaque after anti‐microbial mouthwash use. The authors reported bacterial shifts only for saliva and tongue samples and also that PMA treatment enhanced the observed differences only for the saliva samples, suggesting that the bacterial composition in tongue and plaque samples is not affected by the DNA from dead cells, whereas that in saliva samples is. The same authors using an in vitro biofilm model, have shown that PMA treatment enhanced the observed differences after chlorhexidine rinse as compared to non‐PMA treated samples (Exterkate et al., 2014). In the field of orthopedic surgery, when PMA in combination to PCR was compared to culture and standard PCR techniques for the detection of residual periprosthetic joint infection, an enhancement of the specificity and sensitivity of 89% and 79% respectively, was reported in PMA‐treated samples (Askar et al., 2019).

Our results further showed a similar pattern of declining bacterial counts after treatment with both methods. The fact that bacterial DNA was still present is not surprising. After loss of viability, DNA is slowly degrading and amplifiable DNA may be present long after treatment (Brundin et al., 2010). This could explain the detection of periodontal pathogens after effective periodontal treatment in several studies, despite the clinical resolution of periodontitis (Cionca et al., 2010; Mombelli et al., 2017) and in patients in maintenance care (Moëne et al., 2010; Müller Campanile et al., 2015). However, for some bacteria (mainly Td and Pm) we found smaller concentrations when plaque and saliva samples were pre‐treated with PMA. The course of declining bacteria after treatment, was more evident for the group having received the antibiotics as adjunct to mechanical treatment (Group 2), as all 5 bacteria decreased significantly in plaque samples with both methods. For the saliva samples only Pm did not change from baseline to 1 month in this group. Furthermore, the percentages of reduction of each bacteria presented only few discrepancies between the 2 methods: for the group in maintenance care (Group 3) the levels of Pm in plaque decreased by 67% when samples were pre‐treated with PMA versus 5.5% in samples without PMA. In the saliva samples, the percentage of reduction was higher in the non‐ PMA treated samples for Pg (44% vs. 20% in PMA‐treated samples) and lower for Pi (34% vs. 64%). Pm and Pi are important periodontal pathogens of the orange complex (Socransky et al., 1998). Recently, Pm has been found to be associated with the enhancement of Pg virulence properties, since it can induce the production of gingipains (Neilands et al., 2019) as well as to have important synergistic effects on biofilm formation (Horiuchi et al., 2020).

In vitro, the addition of PMA after antibiotic treatment resulted in a reduction of 50% of viable Aa while conventional qPCR resulted in a minor reduction of 2% (Polonyi et al., 2013). For Pg, the reduction was around 30%–50% and was faster than that of Aa (after 24 h vs. after 72 h). At 72 h, the detection level with the PMA‐qPCR dropped to almost 0% while with qPCR remained at 60%.

Although promising results, Exterkate et al. (2014) based on a saliva‐derived polymicrobial biofilm model, suggested that PMA should be used with caution as it can affect the ability of cells' growth. The possibility of PMA to enter viable cells cannot be excluded, thus rendering the product not 100% selective. This could also happen in the presence of excess PMA which can be deactivated after interaction with water under exposure to intense light (Nocker et al., 2006). In addition, several factors, such as the combination of dye exposure temperature and dye exposure time should be taken in consideration as they can influence the signals from membrane‐compromised cells and resulting in false‐positive signals (Nkuipou‐Kenfack et al., 2013). A recent multicenter study highlighted some criticalities linked to the PMA molecule, like the possible loss of efficiency and a limit to discriminate the living from dead bacteria, especially when the number of dead cells is very low (Scaturro et al., 2016).

Flow cytometry is another technique allowing the analysis of cell viability, cell vitality and the status or stage of growth cycle (Kennedy & Wilkinson, 2017). It is useful for detecting not only bacterial counts but also other cell populations like epithelial cells and lymphocytes (Aps et al., 2002; Orbak et al., 2003). This technique is fast and can be performed on samples originating not only from clinical samples but also from food and water (Kennedy & Wilkinson, 2017). In fact, flow cytometry has been used in analysis of saliva samples for the quantification of bacterial count in relation to gingivitis (Aps et al., 2002). However, this technique is sensitive and requires that the examined bacterial population is both viable and culturable which limits detection to a specific physiological state (Harkins & Harrigan, 2004). Thus, it would be interesting to compare flow cytometry with PMA/qPCR for analysing subgingival plaque samples after periodontal therapy.

The current study was not aimed at determining the efficacy of non‐surgical periodontal therapy or the benefits of the adjunction of antibiotics for the treatment of periodontitis. The results clearly show the microbiological benefits of both chemical and mechanical treatment when assessed by both techniques. However, the rationale of the present study, was whether PMA treatment has an effect on the measured bacterial composition, resulting in a more accurate way of evaluating the bacterial load, as only the viable bacteria are detected. With the addition of PMA, the microbiological load that was detected was consistently smaller, since only the live bacteria were detected, whereas with the PCR method alone all bacteria present in plaque‐dead/compromised and live bacteria‐ were detected. Although the cultural method is still the “gold standard” for the maintenance of bacterial viability, it is an expensive, and time‐consuming intensive procedure. On the other hand, the PCR method, is a rapid and easy procedure for the detection and quantification of pathogenic bacteria, but it has the disadvantage to detect DNA from both dead and alive bacterial cells. Our results suggest that pre‐treatment of samples with PMA can at least in part, close this gap: it is an easily applied step added to the classical qPCR that removes DNA from non‐viable cells, thus giving more accurate information on the presence of viable bacterial load and the response to periodontal treatment. However, more validation clinical studies involving more oral bacteria should be carried out before routine use of PMA as adjunct to the q‐PCR method.

## CONFLICT OF INTEREST

The authors declare no conflict of interest.

## AUTHOR CONTRIBUTIONS

*Conception, design of the study*: Catherine Giannopoulou, Andrea Mombelli. *Acquisition of data*: Maria Sereti, Alkisti Zekeridou. *Analysis and interpretation of the data*: Maria Sereti, Alkisti Zekeridou, Jose Cancela, Andrea Mombelli, Catherine Giannopoulou. *Drafting of the manuscript*: Catherine Giannopoulou, Maria Sereti. *Critically revising the manuscript*: Alkisti Zekeridou, Andrea Mombelli, Jose Cancela. *Final approval of the manuscript*: Maria Sereti, Alkisti Zekeridou, Jose Cancela, Andrea Mombelli, Catherine Giannopoulou.

## Data Availability

The data that support the findings of this study are openly available.
